# Transcriptional network dynamics in early T cell development

**DOI:** 10.1084/jem.20230893

**Published:** 2024-08-21

**Authors:** Boyoung Shin, Samantha J. Chang, Brendan W. MacNabb, Ellen V. Rothenberg

**Affiliations:** 1 Division of Biology and Biological Engineering California Institute of Technology, Pasadena, CA, USA

## Abstract

The rate at which cells enter the T cell pathway depends not only on the immigration of hematopoietic precursors into the strong Notch signaling environment of the thymus but also on the kinetics with which each individual precursor cell reaches T-lineage commitment once it arrives. Notch triggers a complex, multistep gene regulatory network in the cells in which the steps are stereotyped but the transition speeds between steps are variable. Progenitor-associated transcription factors delay T-lineage differentiation even while Notch-induced transcription factors within the same cells push differentiation forward. Progress depends on regulator cross-repression, on breaching chromatin barriers, and on shifting, competitive collaborations between stage-specific and stably expressed transcription factors, as reviewed here.

## Teaser

Multipotent blood progenitors begin T cell development in the thymus, activating a multistep gene regulatory network. This review explores how the dynamic interplay between Notch signals, Notch-induced factors, and progenitor-associated factors control T-development progression by synergizing and opposing each other’s action.

## Introduction

Early thymic T cell development orchestrates a one-way transition between two states, from a multipotent state to a T-lineage committed state (Hosokawa and Rothenberg, 2021; Li et al., 2010a, 2010b; Yui et al., 2010). It entails both the installation of the T cell identity program (activation) and the successful shutdown of plasticity toward non-T cell fates (repression). Profound switches in gene expression programs, chromatin accessibility profiles, demarcation of active and repressive histone marks, and three-dimensional (3D) chromatin architectures all occur during this cell type conversion event (Belyaev et al., 2012; Hu et al., 2018; Isoda et al., 2017; Yui and Rothenberg, 2014; Zhang et al., 2012b; Zhou et al., 2019). Intriguingly, the speed at which this conversion occurs can vary greatly, with a major slowdown between the process in the fetal thymus and the corresponding events in the postnatal thymus (Lu et al., 2005; Ramond et al., 2014). Thus, even when the regulatory drivers of T cell commitment are defined, the responses to them may be governed by variable sets of rules.

Years of research by many groups have identified a group of transcription factors (TFs) that are distinctively active in the T cell lineage. TCF-1 (encoded by the *Tcf7* gene), GATA-3, and the zinc finger TF Bcl11b, working together with the more broadly expressed basic helix-loop-helix (bHLH) E proteins, E2A (*Tcf3*) and HEB (*Tcf12*), and Runx family TFs and their common partner CBFβ are indispensable within the thymus to generate the precursors of all or nearly all T cell subsets (Bain et al., 1997; Barndt et al., 2000; Del Real and Rothenberg, 2013; Engel et al., 2001; Guo et al., 2008; Hattori et al., 1996; Li et al., 2010a, 2010b; Scripture-Adams et al., 2014; Weber et al., 2011; Zhou et al., 2022). They act under the influence of Notch signaling to activate the genes essential for T cell identity. In addition, Ikaros (*Ikzf*) family factors, Myb, Gfi1, and a shifting set of ETS (E26 transformation-specific) family factors play roles at multiple developmental timepoints (Anderson et al., 1999; Arenzana et al., 2015; Bender et al., 2004; Chari and Winandy, 2008; Eyquem et al., 2004; Lieu et al., 2004; Oravecz et al., 2015; Phelan et al., 2013; Yu et al., 2010; Yucel et al., 2003; Zamisch et al., 2009). The T cell precursors thus generated then undergo T cell receptor (TCR)–dependent selection and later diversify through expression of additional factors including Th-POK (*Zbtb7b*), T-bet and its relative Eomesodermin (*Eomes*), RORγt, Foxp3, and/or PLZF (*Zbtb16*), together with contributions by different activation-driven TFs, especially STAT family factors, basic leucine zipper factors such as Batf and Maf subfamily members, and interferon-response factors (Alonzo et al., 2010; Ciofani et al., 2012; Egawa and Littman, 2008; He et al., 2000, 2005; Jenkinson et al., 2007; Kreslavsky et al., 2009; Muroi et al., 2008; Narayan et al., 2012; Savage et al., 2008; Wang et al., 2008, 2011; Xi et al., 2006; Yosef et al., 2013; Zhao et al., 2022b). However, the shared origin of all these subsets of T cells springs from the actions of TCF-1, GATA-3, Runx factors, the E proteins, and Bcl11b, collaborating under the influence of strong Notch pathway signaling in the thymus. All of these factors also work later in thymic repertoire selection (Jones-Mason et al., 2012; Kojo et al., 2017, 2018; Steier et al., 2023; Steinke et al., 2014; Xiong et al., 2013) and go on to play roles in the peripheral T cells as well (Bevington et al., 2017; Zhong et al., 2022).

T cell identity itself is stable once established during early thymocyte stages. An intrinsic core of T cell defining gene expression (*Cd3g*, *Cd3d*, *Cd3e*, *Cd247* [also known as TCRζ], *Itk*, *Lck*, *Zap70*, and *Bcl11b*) persists throughout all of the dynamic responses of mature T cells in their in vivo environments (Mingueneau et al., 2013; Yoshida et al., 2019), even despite the potential of memory T cells to undergo virtually unlimited rounds of proliferation (Soerens et al., 2023). Notably though, the persistent expression of these core T cell identity genes does not entirely depend on the persistence of the original TFs responsible for turning on these genes during early T cell development. In fact, some of these original TFs are downregulated to near undetectability in some mature T cell subsets, e.g., TCF-1 and LEF-1 in some subsets of effector T cells, and GATA-3 in many CD8 T cells (Gounari and Khazaie, 2022; Yoshida et al., 2019; Zhao et al., 2022a). Thus, some TF actions in T cell development must be hit-and-run, stabilized later by other factors and/or epigenetic mechanisms yet to be defined.

The central focus of this review is how the initial multipotency epigenetic state can be pushed into a new, T-lineage–promoting configuration, opening new T-lineage–associated enhancers and shutting down progenitor cell enhancers. The basic question we consider is how this process can be coordinated despite taking highly varied lengths of time for the cells to escape from the multipotent progenitor-cell state. Most of the data reviewed come from the mouse system where detailed genetic and cellular manipulations are easier, but key results will be compared with corresponding evidence that has emerged from the human system. Recent evidence fills out the picture of a Notch-driven, feed-forward gene network promoting T-lineage entry, but also indicates that the individual cells starting the T cell program are initially held back in their response to Notch signaling by the continuing operation of stem-progenitor factors. A regulatory struggle between the progenitor-factor network and the Notch-driven T cell gene network is seen to cause a staggered, asynchronous entry of the cells into the T cell program. Key factors affecting the balance between the multipotent progenitor state and the new T-lineage program have now been identified together with a rapidly clarifying picture of the genomic binding patterns that confer their functional linkages within both networks. These factors are implicated as major positive and negative controllers of the speed with which cells launch the T cell program.

## Brief review of early thymic T cell development

### Major stages of early T cell development in vivo

**Figure 1.**
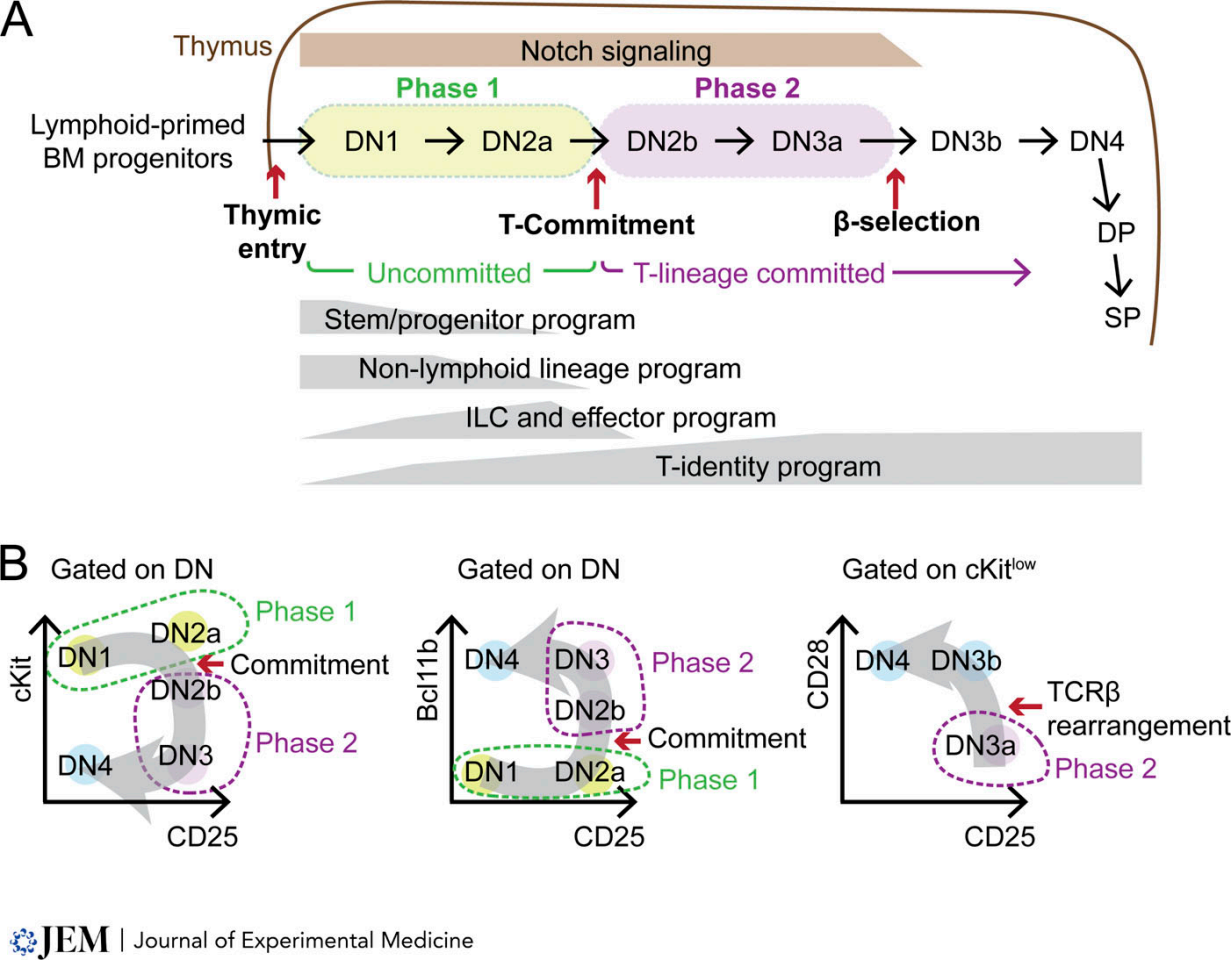
**Early thymic T cell development stages and gene expression programs. (A)** The diagram shows different T cell developmental stages from bone marrow (BM) progenitors entering the thymus and progressing through DN, double positive (DP), and single positive (SP) stages. The focus of this review, Phase 1 (uncommitted) and Phase 2 (T-lineage committed but not yet assembled TCR β) are shown in green and purple bubbles. The instructive Notch signaling strengths are shown in light brown color. The kinetics and amplitude of different gene expression programs are represented in gray boxes at the bottom. **(B)** The informative protein markers utilized to identify different DN stages are shown with flow cytometry plot diagrams. The gray arrows represent developmental progression directions. Critical checkpoints (e.g., T-lineage commitment, β-selection) are also shown.

Thymic T cell development in postnatal mammals begins with small numbers of lymphoid-lineage primed multipotent progenitor cells from the bone marrow that migrate to the thymus for their differentiation (Ezine et al., 1984; Foss et al., 2001; Zietara et al., 2015). These thymus-seeding cells may include both common lymphoid progenitors (CLP) and lymphoid-primed multipotent precursors (LMPP), which converge on the same developmental pathway once in the thymus but differentiate with different kinetics (Benz et al., 2008; Saran et al., 2010). Thymic cortical epithelial cells provide Notch ligands and cytokine signaling that ultimately instruct T cell development while supporting cell proliferation (Besseyrias et al., 2007; Felli et al., 1999; Kyewski, 1987; Prockop et al., 2002; Sambandam et al., 2005). The developmental status of intrathymic T progenitor cells at each stage can be marked by each cell’s transcriptional programs, partially represented by sets of surface protein markers. Initially, thymic pro-T cells are double negative (DN) for CD4 and CD8 and do not express TCR chains because none of the TCR coding loci are yet assembled by recombination. The developmental steps of mouse DN cells encompass DN1 through DN4 stages (DN1, DN2a, DN2b, DN3a, DN3b, and DN4) (Fig. 1 A). These stages are traditionally distinguished by cKit, CD44, and CD25 surface marker expression, along with additional surface markers and TF reporters (e.g., Flt3, HSA, Ly6d, Thy1, CD27, CD28, Bcl11b, etc.) (Ceredig et al., 1985; Godfrey et al., 1993; Hosokawa and Rothenberg, 2021; Kueh et al., 2016; Ramond et al., 2014; Sambandam et al., 2005; Taghon et al., 2006; Teague et al., 2010; Zhou et al., 2019) (Fig. 1 B). Here, we use “DN1” only for the early thymic progenitor subset of CD44^+^ CD25^−^ cells, called ETP, marked as cKit^high^CD44^+^CD25^−^. The cells that have just seeded the thymus are a subset of this DN1 population, usually distinguished by the expression of Flt3.

Upon engaging with the thymic microenvironment, the DN1 and DN2a (cKit^high^CD44^+^CD25^+^) cells begin to execute the T-lineage–specific developmental program, inducing a wave of TFs necessary for T-lymphoid identity programs (discussed in detail below). However, DN1 and DN2a cells are still capable of generating other types of immune cells besides T cells. This does not require any genetic modification (“reprogramming”) of the cells, but simply moving them to Notch ligand-deprived environmental conditions, thus demonstrating their intrinsically “uncommitted” states (Balciunaite et al., 2005; Bell and Bhandoola, 2008; Kueh et al., 2016; Shen et al., 2003; Shortman et al., 1998; Wada et al., 2008; Yui et al., 2010). This plasticity was long known to be gone by the DN3 stage, and it is now clear that lineage commitment to the T cell fate is normally finalized within the DN2 stage, during the DN2a to DN2b (cKit^int^CD44^+^CD25^+^) transition, as pro-T cells from the DN2b stage onward no longer maintain the potential to generate non-T cells (Kueh et al., 2016; Yui et al., 2010). Forced genetic manipulation of DN3 cells can still enable them to switch to alternative fates, and this has been useful in elucidating the functions that underlie or oppose the commitment process (Del Real and Rothenberg, 2013; Franco et al., 2006; Hosokawa et al., 2018a; Ikawa et al., 2010, 2016; Laiosa et al., 2006; Lefebvre et al., 2005; Li et al., 2010a, 2010b; Qian et al., 2019; Steier et al., 2023; Taghon et al., 2007; Ungerbäck et al., 2018); nevertheless, such genetic reprogramming depends on breaking the endogenous regulatory state of the DN3 cells and not on exploring the potentials it naturally affords. During and after T-lineage commitment, DN2b and DN3 (cKit^low^CD44^−^CD25^+^) progenitors establish the T-identity gene expression program (Fig. 1) as an increasing number of T cell fate–promoting TFs is turned on and cells upregulate the genes encoding the main apparatus for TCR signaling: *Cd3g*, *Cd3d*, *Cd3e*, *Lck*, *Lat*, *Itk*, and others (Mingueneau et al., 2013; Zhang et al., 2012b). One key outcome of the T-identity program in DN2b and DN3 stages is the rearrangement and expression of TCR genes, enabled by strong upregulation of *Rag1* and *Rag2* expression from DN2b to DN3a stage.

Pro-T cells on the trajectory to generate conventional αβT cells rearrange the TCRβ coding locus and perform a quality check by inducing apoptosis of the cells that fail to express TCRβ. This quality checkpoint, called β-selection, marks the boundary between DN3a (CD27^low^ CD28^−^ DN3) and DN3b (CD27^high^ CD28^+^ DN3) stages (Taghon et al., 2006; Teague et al., 2010) (Fig. 1 B). Descendants successfully crossing this threshold proliferate extensively, become CD4^+^CD8^+^ “double positive” thymocytes, and undergo TCR repertoire selection. Other T-progenitors at the DN2–3 stages recombine and express genes encoding TCRγ and TCRδ instead and become γδT cells (Capone et al., 1998; Munoz-Ruiz et al., 2017). The decision between γδT and αβT trajectories is a complex one that integrates intrinsic biases due to alternative TF activities together with the cells’ experience of strength of TCR/CD3 complex signaling. Through most of this review, we will treat both αβ and γδT cells as emerging from the same pool of DN1 and DN2a intrathymic precursors, but note that some fetal-specific γδT lineages probably arise from separate prethymic sources (Beaudin et al., 2016; Elsaid et al., 2021; Ikuta et al., 1990).

The DN stages when T cell identity is formed have been intensively studied by many groups to determine the extent and order of gene expression and chromatin state changes (Arenzana et al., 2015; Hosoya et al., 2018; Hu et al., 2018; Oravecz et al., 2015; Yoshida et al., 2019; Zhang et al., 2012b). Because differentiation is coupled with proliferation until the β-selection checkpoint, at steady state, the fraction of DN cells within the DN1 (ETP) stage(s) is minuscule, and it has been important to use single-cell transcriptomics to characterize these early cells fully. In mice (Kernfeld et al., 2018; Sagar et al., 2020; Zhou et al., 2019) and humans (Lavaert et al., 2020; Le et al., 2020; Zeng et al., 2019), a continuum of thymus-induced gene expression changes is seen to begin within the DN1 stage, where a difference is already evident between initial thymus-seeding progenitor (TSP) immigrants and more advanced DN1 (ETP) cells. New genes are upregulated and progenitor legacy genes decrease expression in several stages as the cells enter the DN2a stage and proceed up to β-selection, similarly in mice and humans overall, despite some species differences (Le et al., 2020; Rothenberg, 2021; Taghon and Rothenberg, 2008).

The chromatin accessibility, interaction, and compartment landscapes of DN1 and DN2a cells are remarkably similar to those of hematopoietic stem and multipotent progenitor cells (Hu et al., 2018; Yoshida et al., 2019). However, there is a sharp climax of gene expression change in the DN2a to DN2b transition coinciding with a particularly widespread shift in chromatin accessibility patterns across the genome (Hu et al., 2018; Yoshida et al., 2019) as the chromatin landscape flips from a progenitor-like pattern to one resembling later T-lineage stages. This discontinuity indicates that “commitment” is not just another step in an incremental process but a global regulatory transformation. Therefore, the developmental stages before T-lineage commitment (DN1 and DN2a) are referred to here as “Phase 1” and the stages after T-commitment until β-selection (DN2b and DN3a) are defined as “Phase 2” (Yui and Rothenberg, 2014) (Fig. 1).

### In vivo versus in vitro models

In vivo, thymocytes migrate through a succession of anatomical compartments during their progression from TSP to the committed DN3 stage, and this complex 3D stromal architecture could certainly provide distinctive inductive signals at different stages along the way (Buono et al., 2016; Lancaster et al., 2018; Lind et al., 2001; Love and Bhandoola, 2011). Furthermore, the organization of the fetal thymus when it is first populated is also different from that of the postnatal thymus (Anderson et al., 2006; Holländer et al., 2006). Despite this, however, it is remarkable that the succession of transcriptional states found in vivo is very well replicated by purified hematopoietic progenitors entering the T cell pathway in vitro (Zhou et al., 2022), whether induced by monolayer cocultures with OP9-DLL1 (Schmitt and Zúñiga-Pflücker, 2002) or TSt4-DLL1 stroma (Miyazaki et al., 2005), or with MS5-DLL4 stroma in artificial thymic organoids (Montel-Hagen et al., 2020; Seet et al., 2017). This indicates that a great part of the blueprint for the T cell commitment process is based on cell-intrinsic gene network circuitry operating in broadly permissive conditions of Notch signaling, more than on a precisely choreographed sequence of distinct, extrinsic signals.

While the stromal cocultures generally drive more proliferation in the earliest stages than the thymus microenvironment does in vivo, the gene expression patterns they induce at each DN stage match in vivo stages extremely closely (Zhou et al., 2022). Furthermore, they enable precursor cells to differentiate into T-lineage cells while preserving distinctive intrinsic features of their T cell precursor activities. For example, they faithfully preserve the cell-intrinsic differentiation speed differences between CLP and LMPP precursors (Montel-Hagen et al., 2020; Taghon et al., 2007) as well as the marked differentiation speed differences between early fetal, late fetal, and postnatal precursors (Ramond et al., 2014; Scripture-Adams et al., 2014) (unpublished data) described below. This broad concordance between in vivo and in vitro differentiation is important in practical terms because it has allowed the roles of particular regulators of the T cell developmental process to be measured within rigorously defined developmental time windows, something that is difficult if not impossible in vivo, and has enabled cause and effect relationships and kinetics to be demonstrated directly in real-time.

## T lineage entry dynamics

The molecular events promoting the transition to T-lineage identity occur in the context of rapid cell proliferation, in response to signals from the thymic microenvironment including Notch (throughout Phase 1 and Phase 2) and IL-7 signaling (starting before T-lineage commitment) (Garcia-Peydro et al., 2006; Hare et al., 2000; Huang et al., 2003; Magri et al., 2009; Spolski et al., 2023; Tani-ichi et al., 2013). In vivo in steady state, the observed distribution of offspring cell numbers among each stage, between precursor entry and the generation of T-lineage committed precursors, is affected by the number of cell cycles that thymic progenitor cells spend during each stage and the cell death rate experienced at each stage (Krueger et al., 2017; Manesso et al., 2013; Olariu et al., 2021), not only the rate (or efficiency) at which individual cells differentiate from one stage to the next. This makes it difficult to gauge effects on developmental speed as such from steady-state subset distributions in vivo, as they can be mimicked or masked by compensatory population effects.

When a discrete cohort of cells can be followed in real time, however, both classic work with chimeras in vivo and more recent studies on developmental kinetics in vitro have shown that the developmental transition from Phase 1 to Phase 2 is a slow process, at least in cells from adult mice. Transferring bone marrow–derived progenitors into non-irradiated hosts showed that progenitor cells remained at the DN1 stage for 9–10 days across 7–10 cell divisions (Manesso et al., 2013; Porritt et al., 2003), and cells spent an additional 2–3 days, roughly four more cell divisions, to undergo T-lineage commitment (Olariu et al., 2021). Consistent with high proliferation rates, developing T-progenitors’ single-cell transcriptomes often show that thymocytes in all developmental stages from TSP until DN3a include a full range of cell cycle stages in mice and humans (Lavaert et al., 2020; Park et al., 2020; Zhou et al., 2019, 2022). Thus, these time intervals between developmental milestones each represent multiple cell cycles.

At the clonal level, thymocytes differentiate from TSP to Phase 2 asynchronously. Most dramatically, TSPs in postnatal thymus take much longer to go through the same apparent stages than TSPs in fetal thymus, proliferating more along the way (Lu et al., 2005), even though the overall order of TF changes matches closely, in the transcriptome studies (Belyaev et al., 2012; Kernfeld et al., 2018; Sagar et al., 2020) recently reviewed in detail (MacNabb and Rothenberg, 2023). While this could partly reflect the differences between the fetal and adult thymic stromata in vivo, the cells also reproduce these differences in timing to reach defined phenotypic milestones when differentiating on the “level playing field” of the OP9-DLL1 stromal cocultures, as noted above. Furthermore, even a single cohort of prethymic LMPP precursors or a highly purified subset of intrathymic DN1 (ETP) cells differentiates asynchronously over a fixed time window (Zhou et al., 2019, 2022). When starting with single purified DN1 (ETP) thymocytes from a postnatal mouse, direct live imaging of individual clones over a period of 1–6 days, as well as endpoint phenotyping of individual clones, has shown that different clones differentiate at significantly different speeds from ETP to DN2a and again from DN2a to DN2b (Olariu et al., 2021; Zhou et al., 2019). This asynchrony is evident in real time despite scRNA-seq demonstrating that the cells follow the same trajectories among transcriptional states (Zhou et al., 2019, 2022). For cells crossing the boundary from uncommitted DN2a to committed DN2b, detailed imaging-based pedigree analysis shows that even cells derived from the same clonal precursor differentiate at different speeds (Kueh et al., 2016; Ng et al., 2018). Stochastically timed system behavior is not surprising in the context of mature T cell responses to antigen receptor stimuli (Feinerman et al., 2008; Kakaradov et al., 2017; Richard et al., 2018), but differs markedly from the deterministic timing of well-studied embryonic developmental programs (Clark and Akam, 2016; Peter et al., 2012), which are highly coordinated at 0.2–5 h timescales despite stochastically bursty transcription at much shorter timescales (Berrocal et al., 2020; Bothma et al., 2014). Thus, early T cell development can encompass varying numbers of days and cell divisions while responding to Notch signaling before the cells activate the T cell core program and proceed to lineage commitment.

## Transcription factors regulating cell fates in Phases 1 and 2

### The Phase 1 ground state

**Figure 2.**
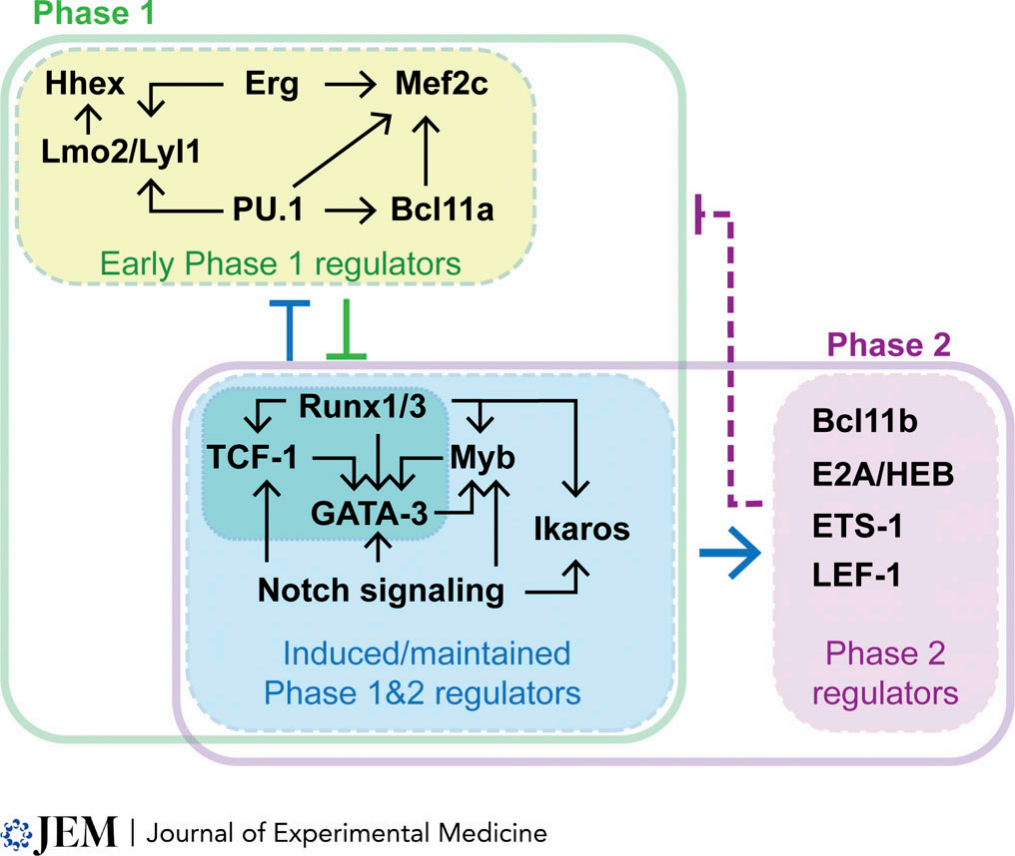
**Distinct groups of TFs shaping the Phase 1 and Phase 2 gene networks.** TFs critically regulating Phase 1 (green) and Phase 2 (purple) transcriptional states are shown in the overview; for detailed relationships, see Fig. 3. TFs with similar expression kinetics are displayed in the same-colored box (green, early Phase 1-expressed TFs; blue, TFs present in both Phase 1 and Phase 2; purple, TFs show high activities in Phase 2). Thin arrows indicate positive (→) or negative (⊣) regulatory inputs between the TFs. Thick arrows represent broad regulatory effects by the sum of TFs’ activities. The dashed purple arrow shows negative inputs toward the early Phase 1 program (green).

**Figure 3.**
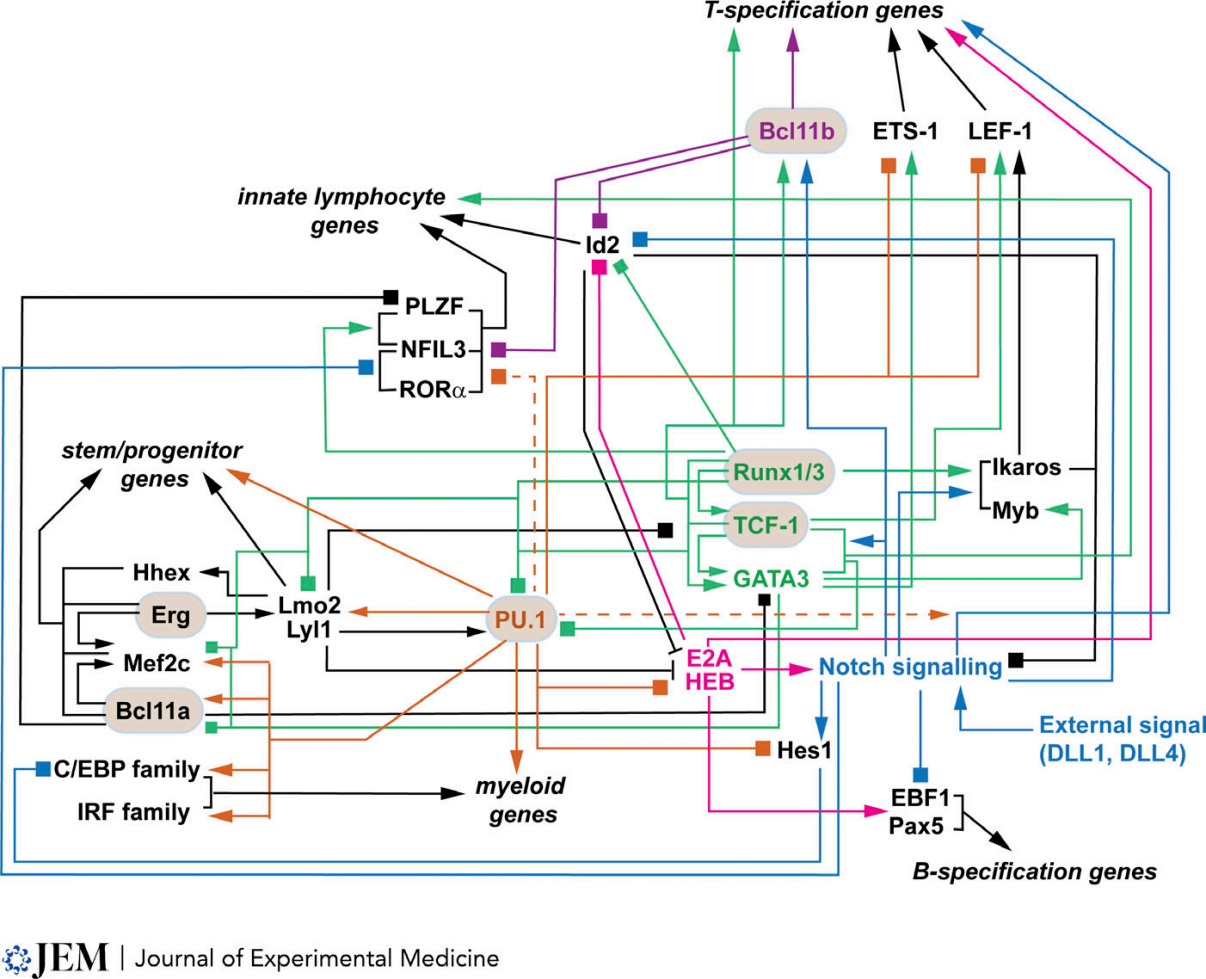
**Gene regulatory network connections within cells transitioning from the uncommitted, thymic precursor stage to a committed T cell stage.** Displayed connections reflect interactions between early Phase 1 regulators (Hhex, Erg, Mef2c, Lmo2/Lyl1, PU.1, Bcl11a), induced/maintained Phase 1 and 2 regulators (Runx1/3, TCF-1, GATA3, Myb, Ikaros, Notch signaling), and Phase 2 regulators (Bcl11b, E2A/HEB, ETS-1, LEF-1), under conditions of Notch signaling. All relationships shown represent functional gene expression impacts of perturbations of individual regulators; curated data from Shin and Rothenberg (2023). Arrows: positive regulation; boxes: negative regulation; bar end: inhibition of DNA binding; broken lines: weaker effects. Colors highlight core subsets of interactions centered around PU.1 (orange), E proteins (magenta), Notch signaling (blue), Runx/TCF-1/GATA3 (green), and Bcl11b (purple). “Speed regulators” discussed in this review are in tan bubbles: Bcl11b, Runx1/3, and TCF-1 have network contributions to both the innate lymphocyte program and the T-specification program, while PU.1 network contributions reach the stem/progenitor, myeloid, T cell specification, and innate lymphocyte programs. PU.1 has additional repressive effects when Notch signaling is reduced. Erg and Bcl11a are related to the PU.1 network, but less is known about their contributions to the other programs.

A large set of TF actions has been shown to underlie gene expression changes in early T cell precursors, defining many connections in a T-lineage gene regulatory network (reviewed by Kueh and Rothenberg [2012]; Longabaugh et al. [2017]; Rothenberg [2019]; Shin and Rothenberg [2023]). Developmental cell fate transition from Phase 1 to Phase 2 is mediated by dynamic changes among gene expression subprograms associated with T-identity, stem and progenitor properties, alternative lineage potentials, and cell proliferation, respectively, all of which are potentially active in cells before commitment. These programs form a modular structure of gene regulatory network subcircuits, in which the expression of a group of genes within each module may be coherently regulated, as presented in detail elsewhere (Shin and Rothenberg, 2023). Fig. 2 offers a low-resolution process diagram of the component gene regulatory modules that slowly shift the cells from the multipotent, Phase 1 state(s) toward increasing T cell character and finally to T-lineage commitment (Phase 2); more detailed aspects are shown in Fig. 3. TFs in pro-T cells initiate, synergize, oppose, stabilize, or repress various subprograms by providing distinct directional inputs into different modules. (For additional details about individual factors, see other reviews [Bao et al., 2022; De Obaldia and Bhandoola, 2015; De Pooter and Kee, 2010; Hosokawa and Rothenberg, 2021; Naito et al., 2011; Shah and Zúñiga-Pflücker, 2014; Yui and Rothenberg, 2014].) Importantly, both the “maintenance” and “switching” of these distinct gene networks in Phase 1 and Phase 2 depend upon the cooperative activities of multiple TFs.

The Phase 1 program is shaped by an ensemble of TFs inherited from the bone marrow progenitor cells. In the early Phase 1 stage, the TFs PU.1 (encoded by *Spi1*), Erg, Lmo2, Lyl1, Hhex, and Bcl11a control gene expression programs and self-renewal properties of the cells (Cleveland et al., 2013; Hosokawa et al., 2018b; McCormack et al., 2010; Thoms et al., 2011; Ungerbäck et al., 2018; Zhou et al., 2022). Binding of the pioneer factor PU.1 (Frederick et al., 2023; Minderjahn et al., 2020; Pham et al., 2013) prominently contributes to the chromatin accessibility landscape in these cells (Ungerbäck et al., 2018). Transcription factors Hoxa9, Meis1, and Mef2c are also expressed in TSPs, while N-Myc (*Mycn*) and Hhex remain active in the later ETP and DN2a Phase 1 cells. All have their expression shut off at different points during progression through T lineage commitment or shortly thereafter (Mingueneau et al., 2013; Yoshida et al., 2019; Zhou et al., 2019). These factors support the expression of genes associated with stem/progenitor programs and sustain intrinsic myeloid potentials. However, their action within pro-T cells is impacted by the strong influence of the thymic microenvironment.

Notch signaling and the Notch-induced repressor Hes1 alter the activities of these legacy TFs, not only by directly regulating their expression levels but also by indirectly promoting or antagonizing their functions selectively, through repressing their partner factors or directly altering the expression of some of their target genes (Canté-Barrett et al., 2022; Chari and Winandy, 2008; De Obaldia et al., 2013; Del Real and Rothenberg, 2013; Franco et al., 2006; Germar et al., 2011; Guo et al., 2008; Ikawa et al., 2006; Laiosa et al., 2006; Romero-Wolf et al., 2020). In parallel, strong Notch signaling inhibits the expression of a key contributor to the innate lymphoid alternative programs, namely Id2, an antagonist of bHLH E proteins (Chea et al., 2016). Notch signaling also sharply reduces the ability of PU.1 to promote myeloid differentiation, at least in part by inhibiting expression of the obligatory myeloid partners of the C/EBP and IRF families, while also inhibiting expression of *Id2*, which could otherwise promote innate lymphoid fates (De Obaldia et al., 2013; Del Real and Rothenberg, 2013; Romero-Wolf et al., 2020; Rothenberg et al., 2019). Thus, while Notch signaling persists, PU.1 at natural levels initially appears to support the proliferation and expression of progenitor genes without undermining T-lineage potential.

### Initiation of T-identity program against retention of stem and progenitor program in Phase 1

This is the Phase 1 context in which Notch signaling induces new regulators, including TCF-1 (encoded by *Tcf7*) and GATA-3, which are essential for initiating the T-identity program in a nonredundant manner (Besseyrias et al., 2007; Germar et al., 2011; Harly et al., 2020; Hattori et al., 1996; Hozumi et al., 2008; Romero-Wolf et al., 2020; Zhou et al., 2022). TCF-1 and GATA-3 are also essential for precursors of innate lymphoid cells (ILCs), emphasizing the root connections between T and ILC programs (Cherrier et al., 2018; Harly et al., 2019; Kasal and Bendelac, 2021; Zhong et al., 2020). Notably, in DN1 thymocytes, these key factors are induced in the same cells that are still expressing the stem and progenitor module regulators (Zhou et al., 2019) (Fig. 2). TCF-1 becomes profoundly important for bone marrow–derived precursors to enter the T-lineage program from the earliest stages, as shown by bulk and single-cell analyses of the short-term impacts of TCF-1 knockouts on gene expression (Germar et al., 2011; Weber et al., 2011; Zhou et al., 2022). TCF-1 positively regulates itself and GATA-3 under Notch instruction, increasing their expression levels as pro-T cells progress from Phase 1 to Phase 2. This positive feedback loop along with regulatory inputs from other TFs bolsters the transcriptional state supporting the T-identity program in developing pro-T cells (Fig. 2) and ultimately promotes progression from Phase 1 to Phase 2.

Notch signaling also enables new, T-lineage promoting functions by TFs that are established already in pre-thymic bone marrow progenitors, such as Runx family TFs (esp. Runx1 and Runx3, “Runx1/3”), Ikaros (encoded by *Ikzf1*), and E proteins (E2A and HEB, encoded by *Tcf3* and *Tcf12* respectively), which are necessary to generate functional T cells. This change occurs through factor–factor interactions by which newly induced factors influence the behavior of the pre-established TFs, recruiting them to new sites and/or changing the local chromatin contexts. Ikzf, Runx, and E protein family members are progenitor-inherited but not typical Phase 1 factors, as their expression is sustained through Phase 2 and their effects on target genes under Notch signaling explicitly enhance the T cell program (Figs. 2 and 3). E proteins appear to be needed for gene regulation in both Phase 1 and Phase 2 based on gene expression changes when they are deleted (Miyazaki et al., 2017; Xu et al., 2013), but the signature gene expression targets of the classic E2A:HEB E protein dimers, like *Rag1* and *Rag2*, are only turned on in Phase 2 (Miyazaki et al., 2011, 2020). Runx binding motifs are among the top three motifs enriched at open chromatin sites in both Phase 1 and Phase 2 cells alike (Shin et al., 2021). However, these are not the same sites in the two phases, and the Runx factors do not play a static role. Instead, Runx factors and Ikaros show remarkable abilities to switch their functional target genes in each T-development stage (Arenzana et al., 2015; Shin et al., 2021). Preliminary evidence indicates that this is true for bHLH factors as well (unpublished data).

The shift has been analyzed in detail for Runx factors. Runx factors have a variety of partners in binding the DNA, frequently partnering with PU.1 in a PU.1 activation domain–dependent way during Phase 1 (Hosokawa et al., 2018b; Ungerbäck et al., 2018) as well as with Ets1 (Goetz et al., 2000; Gu et al., 2000; Wotton et al., 1994; Zhao et al., 2017), TCF-1 (Goldman et al., 2023; Shin et al., 2023), and Bcl11b in Phase 2 (Hosokawa et al., 2018a; Kojo et al., 2018). During the Phase 1 to Phase 2 transition, Runx factors relocate globally from sites enriched for co-binding with PU.1 to new sites enriched for co-binding with TCF-1, E proteins, and/or Bcl11b instead (Shin et al., 2021, 2023). Thus, together Ikzf, Runx, and E protein factors, guided by Notch signaling, dynamically transform existing gene networks to initiate the T identity program despite the constancy of their own expression levels, shifting their functional gene targets through interaction with different binding partners.

### Phase 2: Complete shutoff of multipotentiality and establishment of T-identity

As the cells move into Phase 2, they extinguish the expression of the stem/progenitor-associated TFs, sharply increase their expression of T-lineage genes, and also consolidate their T cell identity by blocking access to the related ILC and natural killer (NK) programs.

Three regulatory changes stand out in the transition from Phase 1 to Phase 2 (Figs. 2 and 3). One is the sudden prominence of a bHLH “E protein” TF family binding motif among the most enriched in open chromatin (Johnson et al., 2018; Shin et al., 2021; Yoshida et al., 2019). This is noteworthy because the E2A protein at least is already fully expressed in Phase 1 cells and HEB increases only three to fourfold. However, the motif evidence indicates that the effective activities of E2A:HEB and E2A:E2A dimers are sharply increased in Phase 2. This may reflect the silencing of the gene encoding an alternative Phase 1 heterodimerization partner, Lyl1, which confers a slightly different DNA binding specificity (Hoang et al., 2016).

Second, although there is a complex population of ETS TF family members expressed in the cells throughout early T cell development, there is a particularly notable shift in family member prevalence from Phase 1 to Phase 2 (David-Fung et al., 2009). Through Phase 1, the ETS family member PU.1 dominates the open chromatin landscape, but as it is downregulated, another ETS factor, Ets1, becomes upregulated and predominant within the cells thereafter. Since PU.1 represents an ETS subfamily with a DNA binding specificity distinct from other ETS factors, there is also a shift in the pattern of ETS sites in the genome that are accessible (Johnson et al., 2018; Shin et al., 2021; Yoshida et al., 2019).

Third, the gene encoding the Bcl11b TF is abruptly induced from a virtually silent state to high activity. Bcl11b rapidly becomes one of the strongest partners of Runx factors in Phase 2, extensively overlapping with Runx in DNA binding sites across the genome as Runx abandons many previous co-binding sites with PU.1 (Hosokawa et al., 2018b; Shin et al., 2021). At select sites, Runx stays but swaps a PU.1 partner for Bcl11b (Gamble et al., 2024). By monitoring a nondisruptive fluorescent protein reporter inserted into the *Bcl11b* 3′-untranslated region, *Bcl11b* upregulation has been shown to coincide very closely with functional commitment at the single-cell level (Kueh et al., 2016). Thus, fluorescent protein Bcl11b reporters have become valuable as an additional criterion with CD25, cKit, and CD44 expression to score those cells that have crossed the Phase 1/Phase 2 boundary (Kueh et al., 2016; Ng et al., 2018; Olariu et al., 2021; Shin et al., 2023).

Gene expression in Phase 2 reflects the activities of all these factors. T-lineage gene expression is strongly dependent on TCF-1, which also trends upward in expression from Phase 1 to Phase 2 (Goldman et al., 2023; Johnson et al., 2018). Both TCF-1 and GATA-3 are required to turn on a large fraction of the distinctive T cell signature genes that become strongly expressed at this stage (reviewed in Shin and Rothenberg [2023]), and the landmark upregulation of *Bcl11b* at the transition to Phase 2 itself cannot occur without prior action of TCF-1 and GATA-3 (Goldman et al., 2023; Kueh et al., 2016; Shin et al., 2023). Bcl11b itself appears to exert two kinds of roles in murine T cells: it contributes positively to the activation of T cell signature genes including the CD3 complex and it also blocks diversion to the NK or ILC fate by inhibiting expression of *Id2* and other innate regulators (Hosokawa et al., 2018b; Li et al., 2010b). This latter repressive role is important because the loss of PU.1 and other Phase 1 factors at this point appears to leave the cells primed to embark on an ILC and/or NK trajectory as well as a T cell trajectory, and Bcl11b blocks this major alternative (Zhou et al., 2022). Bcl11b also has positive regulatory impacts on T-lineage gene targets such as the *Cd3* complex, although it is still uncertain whether this impact is direct (Hosokawa et al., 2018b). In the human system, positive regulatory roles of Bcl11b that promote αβT cell development appear more prominent (Dolens et al., 2020; Ha et al., 2017). The Runx:ETS combination, mostly representing Runx1 and Ets1 in Phase 2, is directly implicated in opening the TCRβ coding loci for rearrangement (Majumder et al., 2015; Zhao et al., 2017). Meanwhile, the strong E protein activity drives both upregulations of the gene cluster that encodes the CD3 components of the TCR and upregulation of the gene cluster encoding Rag1 and Rag2, which will catalyze TCR gene rearrangement (Engel and Murre, 2004; Miyazaki et al., 2020).

Silencing of the Phase 1 genes is due to contributions from multiple Phase 2 factors. Notably, these factors seem to be working as positive regulators of T cell program genes in the same cells where they are also working as negative regulators of the Phase 1 regulators (Figs. 2 and 3). These are intrinsically bifunctional factors, although posttranslational modification may also affect the balance of their activating and repressive activities (Mizutani et al., 2015; Vu et al., 2013; Yamagata et al., 2000; Yamashita et al., 2005; Zhang et al., 2012c; Zhao et al., 2008). TCF-1 itself includes not only transactivating domains but also an intrinsic histone deacetylase domain and a Groucho (Transducin-like Enhancer of Split) corepressor binding domain (Brantjes et al., 2001; Xing et al., 2016), enabling it to repress directly as well as activate gene expression. TCF-1 isoforms using a specific N-terminal domain appear to be required to silence a prethymically expressed GATA factor, GATA-2 (Goldman et al., 2023), which could otherwise transdifferentiate the early T cells toward a mast cell fate (Goldman et al., 2023) (a rare but previously reported alternative [Taghon et al., 2007]). The combination of Runx1 and high-level GATA-3, possibly with an additional stage-specific corepressor, appears to repress PU.1 (*Spi1*) (Hosokawa et al., 2021; Huang et al., 2008; Scripture-Adams et al., 2014). Runx factors additionally inhibit other stem/progenitor TFs, *Lmo2*, *Bcl11a*, *Mef2c*, and *Lyl1* (Shin et al., 2021, 2023). GATA-3 itself appears to be most important for downregulating *Bcl11a*, the stem and progenitor cell-associated paralog of *Bcl11b* (Zhou et al., 2022), as well as PU.1 (*Spi1*). A mixture of active repression and activator withdrawal can also be involved in the extinction of the stem and progenitor cell program.

## Speed control of T cell program entry

### Stochastic speed control by epigenetic mechanisms and TF antagonism

What controls the timing of the Phase 1–Phase 2 shift? TF-target gene relationships have yielded the topology of a gene regulatory network in a static form (Fig. 3), but with the strong roles for factors with activities that cross the Phase 1–Phase 2 borders, the dynamics are especially hard to predict or compute a priori. A direct experimental approach has been crucial to gain mechanistic insight. In vitro differentiation cultures promoting early T-lineage development (Montel-Hagen et al., 2020; Schmitt and Zúñiga-Pflücker, 2002; Seet et al., 2017) make it possible to follow a cohort of defined precursors in real-time through the Phase 1 stages and across the Phase 1→Phase 2 transition. By introducing specific genetic perturbations at defined time points in the culture, re-sorting the treated cells from a desired stage, and returning them to culture for short times, the impacts of individual regulators can be characterized in a stage-specific way in real-time. These methods have been crucial in revealing that the same factor can control different genes at different stages within this process (Arenzana et al., 2015; Goldman et al., 2023; Hosokawa et al., 2021; Romero-Wolf et al., 2020; Shin et al., 2021), and that a given target gene may only respond to a particular factor in a stage-specific way (Kueh et al., 2016; Romero-Wolf et al., 2020; Shin et al., 2021). Such experiments have also shown that a few specific TFs can have strong effects on the absolute speeds of progression of individual cells through the T-lineage specification trajectory.

Phase 1 factors play an active role in restraining T lineage progression speed in the cells that coexpress them with early T cell genes. Acute disruption of PU.1, Bcl11a, or Erg markedly accelerates T-lineage progression of the treated cells in cell-autonomous ways, as shown by single-cell transcriptome analysis of a cohort of cells a few days after the deletion (Zhou et al., 2022). These Phase 1 factors each work through distinct target genes (see below), and they are likely not to be the only Phase 1 factors that restrain the T cell program. For example, in some T-acute lymphoblastic leukemias, elevated levels of other Phase 1 TFs like Lmo2, working as proto-oncogenes, are implicated in the developmentally frozen immature phenotype (Cleveland et al., 2013; Smith et al., 2014; Treanor et al., 2014), and a braking role for Lmo2 at natural levels is supported by our preliminary data as well (unpublished data). Note that this Lmo2-dependent retardation of T-lineage differentiation, per se, need not contradict evidence for even earlier positive effects of Lmo2 on the prethymic precursor compartment, reported recently (Hirano et al., 2021). Lmo2 normally exerts its effects in complexes with SCL (Tal1) or Lyl1, which can also inhibit T lineage differentiative progression despite also playing positive roles in maintaining self-renewal and the viability of the Phase 1 population (de Pooter et al., 2019; Hoang et al., 2016; McCormack et al., 2010, 2013; Zohren et al., 2012). Thus, at least four Phase 1 TFs play integral roles in slowing the rate of T-lineage differentiative progression in the presence of Notch signaling, even if they also promote enhanced proliferation or survival.

On the other side, at least two factors have been shown to accelerate T lineage progression in real time: TCF-1 and Runx factors. As noted above, Phase 1 cells are induced by Notch signaling to express TCF-1 while they are still expressing progenitor genes (Zhou et al., 2019). Total loss of TCF-1 can abort activation of the T cell program in bone marrow-derived precursors (Germar et al., 2011; Zhou et al., 2022) and eliminate their competitiveness even to generate ETPs (Weber et al., 2011). Although in steady state some thymocytes develop in TCF-1–deficient^*-*^ mice (<10% normal numbers), this is probably attributable to compensation by the paralogous factor LEF-1, which is activated to high levels in Phase 2 cells and could be expressed early in a small percentage of Phase 1 cells (Okamura et al., 1998; Weber et al., 2011). Notably, even a modest gain of full-length, wild-type TCF-1 expression (∼3× normal) accelerates progression in real-time from DN1 to DN2 stages (Goldman et al., 2023), and strong forced TCF-1 expression can even bypass the need for Notch signaling for many joint target genes (Weber et al., 2011). Its potency may reflect its competence as a pioneer factor (Johnson et al., 2018). This response is specific to TCF-1. In contrast, although GATA-3 is equally indispensable, the range of GATA-3 levels that are tolerated by pro-T cells is much narrower. Whereas some T-lineage accelerating effects of extra GATA-3 can be seen in human cells (Van de Walle et al., 2016), fetal liver-derived mouse precursors can be severely inhibited from T-lineage progression by supraphysiological GATA-3 levels (Taghon et al., 2007; Xu et al., 2013), even in the same stages when they also abort if GATA-3 levels are ∼3× reduced (Scripture-Adams et al., 2014).

Runx factors act as specific, dose-dependent forward drivers of the T cell program from Phase 1 onward, despite being needed for aspects of gene expression both before and after commitment. Not only is T cell development aborted by combined loss of Runx factors’ activity (Guo et al., 2008; Shin et al., 2021), but also, even a modest elevation of Runx factor activity (∼2×) leads to a notable acceleration of T-lineage differentiation within 2 days. Runx-increased cells then differentiate past each milestone 1–2 days faster than controls through 2 wk of development (Shin et al., 2023). Thus, in the presence of Notch signals, the Runx activity level is a major accelerator of the T cell program. This could seem surprising. In Phase 1 cells, Runx1 physically interacts with PU.1 and co-occupies most PU.1 binding sites, especially those that are found in open chromatin (Hosokawa et al., 2018b; Shin et al., 2021; Ungerbäck et al., 2018). However, from gene expression impacts of perturbation, Runx factors more often oppose PU.1’s activity by restricting the cells’ myeloid potentials and inhibiting other PU.1 activation target genes (Shin et al., 2023). In the same cells, moreover, Runx factors amplify the onset of the T-identity program by upregulating TCF-1 and GATA-3 expression (Guo et al., 2008; Shin et al., 2021, 2023). Subsequently, the combination of Notch signaling, TCF-1, GATA-3, and Runx factors activates the expression of *Bcl11b*, i.e., the landmark of transition to Phase 2 (Kueh et al., 2016), with Runx factors playing a particularly strong dose-dependent role (Kueh et al., 2016; Shin et al., 2023). This indicates that even though Runx levels hardly change from Phase 1 to Phase 2 (Shin et al., 2021), Runx proteins under the influence of Notch signaling actively promote progression to Phase 2 and destabilize the stem/progenitor state starting in Phase 1.

### Combinatorial and epigenetic bases of TF dose dependence

The high dose dependence of the Runx effect is likely to reflect two phenomena: the sensitivity of Runx DNA binding to open and closed chromatin states and the association of Runx with different partner factors in Phase 1 and Phase 2. The likelihood of a TF’s binding to any given site depends on a combination of the affinity it has for the site sequence (“quality” = match to optimal position weight matrix), the accessibility of the site in chromatin, and the concentration of the “free” factor in the cell nucleus. In both the cases of PU.1 and Runx1, site accessibility in chromatin appears to be traded off with site quality and with TF concentration to determine the likelihood of occupancy (Shin et al., 2023; Ungerbäck et al., 2018). Although overall Runx protein levels hardly change from Phase 1 to Phase 2, these results suggest that the amount of relevant “free” Runx factor depends on its titration by different competing factors. As long as PU.1 levels are high, Runx appears to be engaged by PU.1 preferentially with reduced availability for its Phase 2 target sites (and alternative potential partner factors). Any extra Runx1 protein appears to enter the “free” pool. This is then available to enter both accessible, empty Phase 2 sites, and previously closed chromatin sites.

Epigenetic constraint is a significant contributor to the late activation of Runx target *Bcl11b* (Hu et al., 2018; Isoda et al., 2017; Ng et al., 2018; Pease et al., 2021). The initial epigenetic state of the *Bcl11b* regulatory region in prethymic progenitors and DN1 (ETP) cells in vivo is closed, demarcated by repressive H3K27me3, and sequestered in (inactive) compartment B (Hu et al., 2018). ChIP-seq evidence implies that this epigenetic state slows responses to physiological levels of TCF-1, GATA-3, and Runx factors by limiting their binding to *Bcl11b* regulatory elements (Goldman et al., 2023; Shin et al., 2021, 2023). It also apparently enforces the requirement for concurrent Notch signaling to turn *Bcl11b* on, which is relieved in Phase 2 (Kueh et al., 2016; Ng et al., 2018), when the regulatory region has been converted to an open, A compartment state (Hu et al., 2018). A similar mechanism may also influence the upregulation of *Ets1* at the Phase 1–Phase 2 boundary (Chandra et al., 2023). However, despite the abilities of closed chromatin states to steepen the barrier for TF binding, they can evidently be overcome in these cases by modest elevation of Runx family factors (Shin et al., 2023), which enables Runx to bind in previously inaccessible but high-quality sites. Slightly overexpressed Runx binds precociously to at least 20 previously closed sites in the *Bcl11b* regulatory region to force *Bcl11b* activation much earlier than normal, even before the cells turn off their Phase 1 genes (Shin et al., 2023). Thus, TF dosage titration likely based on TF partner competition plays a strong role in developmental timing even when chromatin state changes are required.

## Coherent and incoherent actions of co-expressed transcription factors in Phase 1 and Phase 2

Changes in TF functional activity, due to the availability of major regulators and switches in their regulatory assignments, are key to driving the Phase 1 and Phase 2 progression as TF-specific inputs are generated for new target genes. However, in addition to the inputs themselves, another layer of mechanism shapes gene network dynamics: each target gene must integrate inputs received from multiple TFs, acting either in collaboration or opposition. Hence, the developmental states and progression rates of pro-T cells are determined by the rules for summing the activities of multiple TFs operating within the same cell. While still incompletely understood, there are hints of interesting patterns in these relationships.

**Figure 4.**
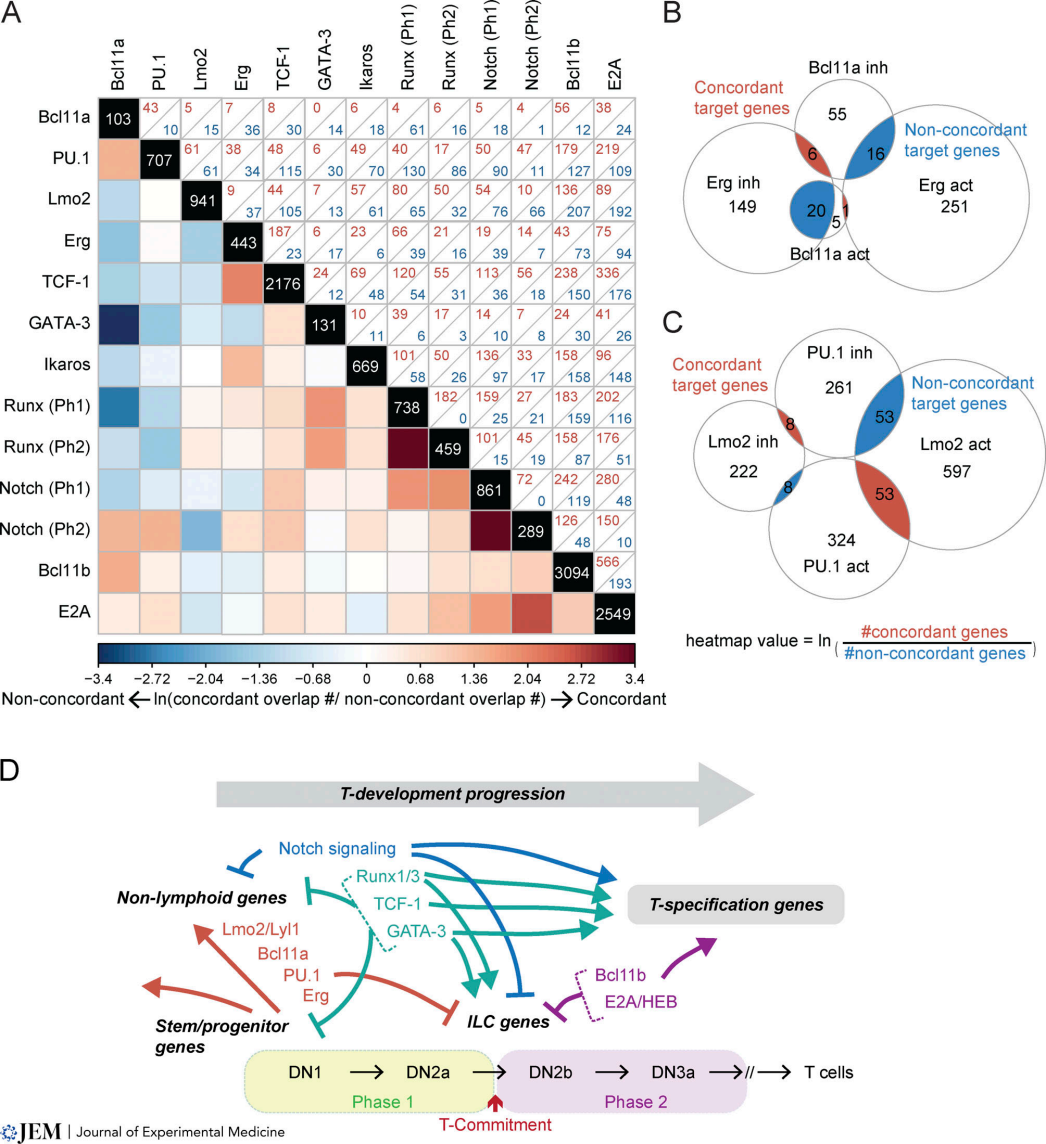
**Converging and opposing actions of TF pairs on shared target genes. (A)** The heatmap illustrates converging (more red) or opposing (more blue) activities of two TFs on their common targets as a natural log of the ratio between numbers of genes that are regulated concordantly (both TFs activate or both inhibit) versus non-concordantly (one activates and the other inhibits). Zero counts were replaced with 0.5 for logarithmic transformation, and natural log ratio values are shown from −3.4 to 3.4. Numbers in diagonal boxes display the total numbers of target genes for each TF. Upper right boxes enumerate joint targets with concordant (red) or non-concordant (blue) effects. Targets were determined in these studies: Zhou et al., (2022) for Bcl11a, PU.1, Erg, TCF-1, GATA-3; Ungerbäck et al. (2018) for PU.1; Hirano et al. (2021) for Lmo2; Arenzana et al. (2015) for Ikaros; Shin et al. (2021) and Shin et al. (2023) for Runx1 and Runx3 (Phase 1 DEGs include both gain-of-function and loss-of-function targets); Romero-Wolf et al. (2020) for Notch; Hosokawa et al. (2018b) for Bcl11b; and Miyazaki et al. (2017) and Xu et al. (2013) for E2A. **(B and C)** Area-proportional Venn diagrams provide examples of how TFs converge or oppose each other’s gene regulation and how the heatmap values are calculated. **(B)** Erg and Bcl11a: non-concordant target genes include *Lmo2*, *Mef2c*, *Dntt*, *Egfl7* (Erg-inhibited, Bcl11a-activated), *Nrgn*, *Gzmb*, *Thy1*, and *Cdkn1a* (Erg-activated, Bcl11a-inhibited). **(C)** PU.1 and Lmo2: non-concordant target genes include T- and ILC-associated genes such as *Tcf7*, *Zbtb16*, *Pou2af1*, *Gzma*, *Cxcr5* (PU.1-inhibited, Lmo2-activated), and *Cd24a* (PU.1-activated, Lmo2-inhibited). **(D)** Summary: T cell program emerges from a balance between T-specification and progenitor factors; roles of specific regulators distinguish between T and ILC programs.

One surprise is that Phase 1 TF roles are often non-concordant, despite examples of Phase 1 factors positively regulating each other and coexpression of Phase 1 factors in the same cells (Zhou et al., 2019). TFs predominantly expressed in early Phase 1 (PU.1, Lmo2, Lyl1, Mef2c, Bcl11a, and Erg) share many common target genes that are non-redundantly controlled by at least two of the factors (Figs. 3 and 4). However, although acute disruption of *Spi1* (encoding PU.1), *Bcl11a*, or *Erg* accelerates early T cell development as noted above, each perturbation results in the pro-T cells shifting to somewhat different developmental trajectories. This suggests that these factors each slow down T-lineage developmental speed by targeting distinct gene expression programs (Zhou et al., 2022). In fact, the common target genes shared simply between two stem/progenitor TFs are often inhibited by one TF while activated by the other TF (Fig. 4 A). For instance, Bcl11a and Erg divergently regulate key genes involved in normal ETP programs (Fig. 4 B), and PU.1 and Lmo2 disagree in their regulation of T- and ILC-associated genes (Cleveland et al., 2013; Hirano et al., 2021; McCormack et al., 2010; Ungerbäck et al., 2018; Zhou et al., 2022) (Fig. 4 C; compiled in Shin and Rothenberg [2023]). Thus, instead of collaborating generally, these Phase 1 regulators have many non-concordant roles even in the multipotent progenitor state, and the Phase 1 state under the influence of Notch signaling may not be intrinsically stable.

In contrast to this Phase 1 discordance, the TFs supporting the T-identity programs have preponderantly concordant effects on common target genes. Runx and GATA-3 effects are highly concordant on their shared targets, TCF-1 and Runx factors collaboratively support Notch target genes in Phase 1, and Notch-mediated gene regulation is further strengthened by the activities of E proteins, especially in Phase 2. This agrees with previous evidence that E proteins are needed to sustain Notch expression itself (Pereira de Sousa et al., 2012; Yashiro-Ohtani et al., 2009). Many core T-lineage signature genes receive positive inputs from more than two regulators non-redundantly (Fig. 3). Interestingly, the regulatory elements of some of these T-identity genes are located within genomic regions undergoing developmental 3D chromatin and local chromatin state changes (Hu et al., 2018; Isoda et al., 2017; Johnson et al., 2018; Wang et al., 2022; Yoshida et al., 2019). An attractive hypothesis is that combinatorial actions of multiple TFs may be needed to reconfigure 3D architecture and local chromatin signatures for imprinting the T-identity program.

TFs supporting T-lineage signatures play a pivotal role in repressing the progenitor program and non-T-lineage programs, in part by antagonism with the actions of PU.1, Lmo2, Bcl11a, and Erg (Zhou et al., 2022). As already noted, Runx factors exert positive and negative regulatory effects that broadly oppose those of PU.1, and they also predominantly oppose the effects of Bcl11a and Lmo2. GATA-3 and Bcl11a particularly oppose each other. While TCF-1 has many effects concordant with those of Erg, it too is strongly opposed to PU.1, undermining the stemness and non-T-lineage gene networks (Fig. 4 A).

As expected, effects that are directly Notch-responsive and stem/progenitor factor effects also show mutual opposition overall (Cleveland et al., 2013; Del Real and Rothenberg, 2013; Hirano et al., 2021; Hosokawa et al., 2018b; McCormack et al., 2010; Romero-Wolf et al., 2020; Zhou et al., 2022). However, the stem/progenitor regulators do not always work against the Notch-mediated gene regulation. In the presence of Notch signals, PU.1 inhibits ILC and NK programs (Champhekar et al., 2015; Zhou et al., 2022), which can also be inhibited by Notch signaling (Chea et al., 2016; Schmitt et al., 2004), while it works positively on multiple genes that are also positively regulated by Notch signaling, e.g., *Cd34*, *Cd24a*, *Cd5*, *Cd3g*, and *Dnmt3b* (Romero-Wolf et al., 2020; Ungerbäck et al., 2018) (Fig. 4). Thus, in Notch signaling conditions, PU.1 can play a supporting role to suppress alternatives to T cell development and may even use its own pioneering activity to prime cells for aspects of the T cell program (Gamble et al., 2024). Future studies can sharpen our understanding of how other TFs inherited from bone marrow progenitors adapt their functions as cells transition from prethymic progenitor stages to intrathymic Phase 1 stage.

Although TCF-1, GATA-3, Runx, Ikaros, Bcl11b, and E proteins all participate in upregulating T-lineage signature genes, they do not always concur: genes associated with the ILC programs, cytokine and chemokine signaling, cell proliferation, and cell survival-related genes can be divergently regulated (Shin and Rothenberg, 2023). TCF-1 and Runx factors differentially regulate environment-sensing and cell survival programs. Importantly, key ILC program genes activated by TCF-1, GATA-3, and Runx factors are inhibited by Notch, Bcl11b, and E proteins, refining the T-lineage fidelity of Phase 2 states (Fig. 4 D). Thus, TFs and Notch signaling work together toward activating the common T-lineage signature genes, yet their counter-actions trim and finalize the Phase 2 network (Fig. 4 D). Taken together, the coherent or antagonizing actions of co-existing TFs on shared target genes mediate the exit of the Phase 1 state and establish the Phase 2 state, and their dynamics contribute to the transitioning speed control.

## In vivo implications

### Environmental influence

Most of the results discussed above come from a deliberately simplified system, where cohorts of T cell precursors from TSP to committed DN3 stages are purified and tracked in real-time in a fixed, “level playing field” in vitro environment with ample Notch ligands, IL-7, and Flt3 ligand cytokines added, and some Kit ligand and Cxcl12 made by the stroma. They establish a baseline for understanding the intrinsic components of differentiation-promoting circuitry. In vivo, in contrast, the cells traverse a variety of intrathymic environments that likely present gradients of cytokines, chemokines, and Notch ligands (Lind et al., 2001; Love and Bhandoola, 2011), and also varied levels of competition from other T cell precursors. These extrinsic factors can be potent at modulating T-lineage output. For example, competition among DN thymocytes for IL-7 can determine niche availability, and hypercompetition for Notch ligands can effectively cut off the Notch signal for many (Prockop and Petrie, 2004; Visan et al., 2006). However, it is interesting that cytokine effects can be mostly the impact of population dynamics effects (proliferation, death) rather than effects on developmental progression speed per se. When *Stat5a* and *Stat5b*, encoding the major signaling factors activated by IL-7/IL-7R, were simultaneously acutely deleted in Phase 1 T cell precursors with viability support (Spolski et al., 2023), strong gene expression changes were seen, but they were almost completely orthogonal to the genes changing expression between Phase 1 and Phase 2 normally. The Stat5a/Stat5b regulon, at least, appeared to be affecting survival genes primarily as a distinct process from developmental progression itself (Shin and Rothenberg, 2023).

Competition between longer-resident and more recent cohorts of developing T cells may still contribute to developmental speed and fidelity in other ways. Compelling evidence suggests that in situations where the influx of new precursors to the thymus is too low, DN cells may “dawdle” in supportive niches too long and this may foster the slow outgrowth of premalignant and malignant cells (Martins et al., 2012, 2014; Peaudecerf et al., 2012). Much remains to be learned about the pathway through which this transformation occurs.

### Implications for oncogenesis

The network architectures reviewed here make the issue of malignancy inevitable. While the full discussion is beyond the scope of this article, the same regulatory factors that here play the stem or progenitor roles in the Phase 1 gene regulatory network are powerful proto-oncogenes associated with T-acute lymphoblastic leukemias (T-ALL), especially the devastating subtype, ETP-ALL (Aster et al., 2011; Baldus et al., 2006; Canté-Barrett et al., 2022; Haydu and Ferrando, 2013; Hoang et al., 2016; Homminga et al., 2011; McCormack et al., 2013; Smith et al., 2014; Soulier et al., 2005; Treanor et al., 2014; Zhang et al., 2012a). More advanced T-ALLs have a different phenotype. Normally, Notch signaling is sharply downregulated during β-selection as pre-TCR signaling takes over. In most T-ALLs, a major etiology appears to be mutations causing prolonged, ligand-independent hyperactivity of Notch at the DN3 stage into β-selection, enabling an explosive combination of pre-TCR and Notch-driven growth. Selection pressure for uncontrolled Notch signaling may come from aberrant expression of single E protein antagonists like SCL (Tal1) or its partner Lmo2, which would otherwise reduce Notch1 expression. The impact E protein inhibition would have can be seen in the linkages of Fig. 3, but these later-type T-ALLs are mostly post-commitment malignancies. In contrast, ETP-ALL often lacks Notch hyperactivation but involves sustained expression of a broad range of Phase 1 regulators that would normally be shut off in the late DN2 stage. This broad retention of a Phase 1 regulatory state also maintains some myeloid potential for the cells, despite the fact that some ETP-ALLs appear to express some TCR complex components at the same time, a hallmark of postcommitment stages. Thus, ETP-ALL appears to be a central failure of the termination of Phase 1 as a whole, either due to primary failed repression or due to actual retrograde differentiation. Any of the links in the gene regulatory network relating Phase 1 and Phase 2 regulators (Figs. 3 and 4) could be a point of vulnerability for developing T cells. In fact, there is still much to learn about the epigenomic processes that complete Phase 1 regulator silencing and make it as safely irreversible as it normally is.

## Conclusion

Lineage specification to the T cell fate requires robust induction of the T-identity programs while establishing complete repression of other lineage potentials and progenitor programs, but only with the correct timing. To achieve this goal, intrathymic progenitor cells utilize multiple mechanisms, including 3D chromatin reconfiguration, histone modification changes, and chromatin accessibility rewiring. Importantly, multiple TFs are needed to shape the T cell gene networks, each by providing distinct inputs to different gene regulatory modules.

The gene networks of developing pro-T cells are not initially stable but develop intrinsic antagonizing forces that can destabilize the cell state. During the Phase 1–Phase 2 progression, TFs supporting the stem/progenitor programs versus TFs inducing the T-identity program often oppose each other by directly inhibiting each other’s expression levels, by affecting TF DNA binding site choices, and/or by neutralizing the expression of each other’s target genes. However, cross-repression is weak enough so that these factors can coexist in the same cells through multiple cell divisions. This tug-of-war influences the developmental state of pro-T cells as well as the Phase 1 to Phase 2 transition rate.

Under this picture of a tug-of-war between the progenitor state and T cell state, it is easy to ascribe the role of developmental speed controllers solely to the program-specific TFs, i.e., progenitor program TFs (Bcl11a, Erg, PU.1) versus T cell–related TFs (TCF-1, Bcl11b). However, to conceptualize developmental kinetics simply as a series of switches whereby progenitor-specific TFs are spontaneously eliminated and T cell–specific TFs are spontaneously activated turns out to be an oversimplification. Kinetic influence is also exerted by the TFs that can exist within either program (especially Runx, but possibly E proteins as well), while even the canonical “program-specific” TFs have network relationships that reach outside of the one-dimensional progenitor/T cell axis. The TFs that bridge multiple lineage-specific programs are also crucial for tipping the balance toward one state over another. The dosage-dependent, lineage-specific, and stage-specific roles of these factors depend on TF protein–protein interaction parameters and quantitative aspects of chromatin state transition requirements, which still remain to be measured. Rather than pinpointing a single bottleneck event, to understand developmental kinetics will be to understand the slow unbalancing act that emerges from the competitive interplay of TFs with distinct partner factors and the chromatin thresholds that need to be breached to gain access to new targets, making gene dosage critical.
